# Identification of initial sites of SIV rebound after treatment cessation

**DOI:** 10.21203/rs.3.rs-6814218/v1

**Published:** 2025-07-11

**Authors:** Louis Picker, Brandon Keele, Afam Okoye, Taina Immonen, Benjamin Varco-Merth, Derick Duell, Candice Nkoy, William Goodwin, Shelby Hoffmeister, Emek Kose, Andrew Conchas, Charles Goodman, Christine Fennessey, Agatha Macairan, William Bosche, Randy Fast, Christopher Homick, Michael Hull, Kelli Oswald, Rebecca Shoemaker, Lorna Silipino, Jorden Welker, Jeremy Smedley, Caralyn Labriola, Michael Axthelm, Jacob Estes, Dan Barouch, Jeffrey Lifson

**Affiliations:** Oregon Health & Science University; Frederick National Laboratory for Cancer Research; Oregon Health and Sciences University; Frederick National Laboratory for Cancer Research; Oregon Health and Sciences University; Oregon Health and Sciences University; Oregon Health and Sciences University; Oregon Health and Sciences University; Oregon Health and Sciences University; Frederick National Laboratory for Cancer Research; Frederick National Laboratory for Cancer Research; Frederick National Laboratory for Cancer Research; AIDS and Cancer Virus Program, Frederick National Laboratory for Cancer Research; Frederick National Laboratory for Cancer Research; Frederick National Laboratory, Leidos Biomedical Research, Inc.; Frederick National Laboratory, Leidos Biomedical Research, Inc.; Frederick National Laboratory for Cancer Research; Frederick National Laboratory; SAIC Frederick, Inc.; National Cancer Institute; Frederick National Laboratory for Cancer Research; Frederick National Laboratory for Cancer Research; University of Louisiana at Lafayette; Oregon Health and Sciences University; Oregon Health & Science University; Oregon Health & Science University; Beth Israel Deaconess Medical Center; SAIC Frederick, Inc., NCI Frederick

## Abstract

Antiretroviral therapy (ART) suspends HIV replication, but virus persists and rebounds after ART discontinuation. Although much is known about the persistent viral population, the tissue origin(s) and the earliest viral dynamics of post-ART viral rebound remain obscure. Here, using barcoded SIVmac239 in rhesus macaques (RMs) and extensive necropsy tissue sampling on ART and early post ART, we defined the spectrum of low-level barcode-specific viral RNA expression in tissues during ART and then assessed initial clonal rebound by identifying barcodes in individual tissues that exceeded this distribution limit (“outliers”). Eight such outlier barcodes were identified in 4 of 11 aviremic (<1 copy/mL) post-ART RM, with 16 additional outliers in 5 of 6 post-ART RM with low viremia (5–30 copies/ml). Nine of these 16 barcodes were also identified in rebound viremia, confirming specific tissues as rebound origin sites. An RM with post-ART viremia of 4700 copies/mL showed 8 outlier barcodes that ranged from a single site to anatomically discontinuous, multi-site spread. Among all identified outlier barcodes, 27 were determined to reflect rebound origins, of which 96% were in the gastrointestinal (GI) tract (26%) or GI-tract-draining lymphoid tissues (70%). These results indicate that distinct tissue sites differentially restrict/promote post-ART viral rebound, with potential therapeutic implications for interventions designed to prevent or control these events.

## Main

ART has the capacity to drastically suppress or eliminate ongoing replication of HIV and SIV, yet residual virus-infected cells persist long-term in both humans and RMs and will, with few exceptions, reignite spreading, progressive infection when ART is discontinued^1–5^. The long-term, stable viral population – the rebound competent viral reservoir (RCVR) – that can re-ignite infection post-ART is complex and includes multiple subsets of CD4^+^ T cells, particularly including clonal expansions of CD4^+^ memory T cells, and possibly macrophages, that are widely distributed throughout the lymphoid tissues of the body^2,4,6–10^. To mediate post-ART viremic rebound, RCVR virus must be genetically intact, replication competent and inducible *in vivo*^8,11,12^, but viral rebound is not a simultaneous universal resumption of virus replication from the entire RCVR, but rather begins with an initial oligoclonal outgrowth of virus from a limited number of infected cells that subsequently spreads throughout the body, reseeding systemic infection^4,13,14^. Virus expression, including viral RNA and protein production and even release of intact virions from infected cells, is an ongoing process during ART^15^, because ART acts not by blocking virus expression by already infected cells, but by preventing *de novo* infection of susceptible target cells^1,16^. Together, these observations suggest that local cellular, anatomic and immunologic tissue milieus that support the persistence of the RCVR might also contribute to regulation of post-ART rebound, and that this local regulation restricting and/or promoting viral reactivation and initial viral spread after ART discontinuation represents a potential point of intervention for therapies designed to interfere with the rebound process, either suppressing it or enhancing it to facilitate clearance of persistent infected cells. Despite the potential importance of understanding the clonal origins of post-ART viral rebound, little is known about these early local events in tissues, with previous work primarily focused on matching on-ART/pre-rebound tissue viral clonotypes with those contributing to overt post-ART viremia with variable success^13,14,17–19^.

Identifying rebound origin sites *in vivo* is a daunting objective, given that the RCVR may be found in hundreds of different tissues throughout the body^10,13,20^, any of which potentially represent sites of origin for rebound viremia. Moreover, the initial events leading to rebound are temporally inconsistent, with the time between ART washout and systemic viral spread varying between individuals, making the task of observing initial viral processes even more challenging. Despite recent advances, imaging technology is currently unable to rigorously distinguish the earliest focal clonal outgrowth that would reflect origin sites leading to viremic rebound^21–23^. To identify the initial tissue origins leading to rebound plasma, we used barcoded SIV^24,25^ in a calibrated RM model of SIV infection^4^, including through extended ART suppression and post-ART rebound, along with strategically timed, on- and off-ART necropsies with comprehensive tissue analysis. We first developed and validated criteria for recognizing initial viral rebound and then used this approach to identify tissue sites of initial post-ART local virus expression and spread leading to early viremic rebound and subsequent viral dissemination. Our results indicate that with our approach it is possible to robustly identify the early stages leading to post-ART rebound and that these early processes preferentially occur in GI tract-draining lymphoid tissues.

### Calibrated RM model with barcoded SIV enables detailed, viral clonotypic analysis of tissues to characterize processes leading to post-ART viral rebound

This study was designed to identify the anatomic sites of viral expression and early spread that lead to viremic rebound after ART cessation using a calibrated RM model with barcoded SIV. We have previously shown that intravenous (IV) administration of SIVmac239M (a virus stock containing ~10,000 viral variants of a SIVmac239 infectious molecular clone, each of which contains a unique 34-base “barcode” between the vpx and vpr genes), allows deep sequencing-based genetic tracking *in vivo* over time of distinct individual, phenotypically equivalent viral lineages varying only in their barcode sequences^4,24,25^. When SIVmac239M-infected RM are started on ART at day 9 or 12 post-infection, high level acute phase viremia is consistently observed, followed by rapid and complete viral control, with establishment of a saturated RCVR, and predictable rebound dynamics with 1–2 clonotypic reactivation events per day leading to rebound plasma viremia at a median 7-days post-ART [plasma viral load (PVL) >15 copies/ml]^4^. We hypothesized that individual viral lineages undergoing initial post-ART viral replication and spread would manifest a measurable increase in local tissue barcode-specific cell-associated (CA) vRNA levels since replicating cells can express up to thousands of RNA copies for every DNA provirus^26^.

**Figure 1:**
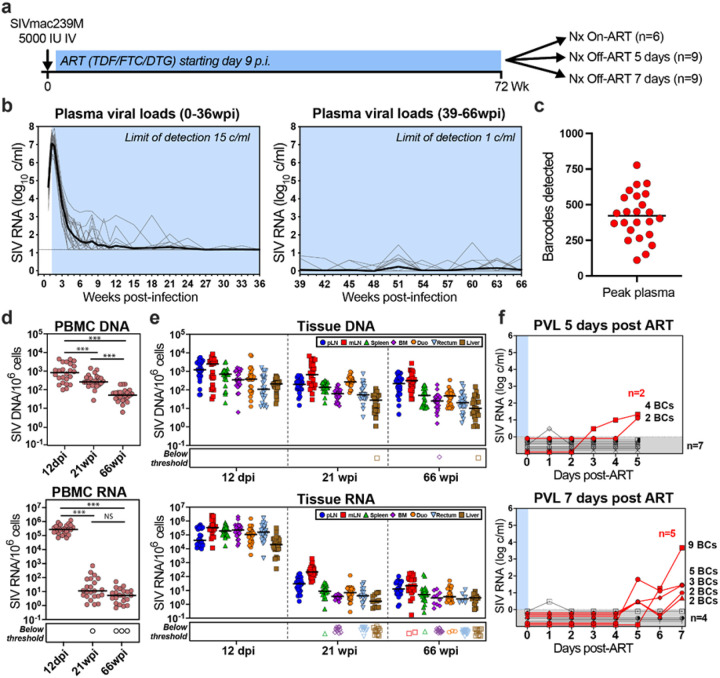
Study schematic and baseline viral measurements. **a**, 24 RMs were infected with barcoded SIVmac239M with daily ART beginning at 9 dpi. RMs were necropsied either on ART (n=6), at 5 days post-ART (n=9) or at 7 days post-ART (n=9). **b**, Blood PVL (assay threshold 15 copies/ml through 36 wpi, then 1 copy/ml thereafter) reveals the characteristic SIVmac239 initial viral growth rate (average 1.6, range 1.3–1.8) and on-ART viral 1^st^ phase decay rates (0.75, range 0.59–0.91). **c**, Peak PVL plasma samples (12 dpi) were used to identify the number of unique barcodes initiating infection per RM (median 422, range 111–649). **d**, Viral DNA and RNA were quantified from PBMCs at 12 dpi, 21 and 66 wpi and were compared between the three time points. Two-sided Wilcoxon signed-rank test. **e**, Both vDNA and vRNA were measured from biopsy tissue samples taken at 12 dpi, and 21 and 66 wpi (peripheral LN-pLN, mesenteric LN-mLN, spleen, bone marrow-BM, duodenum-Duo, rectum and liver). Two-sided Wilcoxon signed-rank test was used to compare each tissue sample for a specific time point. **f**, After 72 wpi, 18 RMs released from ART with daily PVLs obtained, and the detectable rebounding barcode lineages (BCs) identified. 7 of 18 RMs were viremic (≥ 5 copy/ml) at the time of necropsy (2 of 9 from animals necropsied 5 days off-ART and 5 of 9 from animals necropsied 7 days off-ART). There was a total of 27 barcodes detectable in blood at necropsy (mean 3.86; range 2–9).

To test this hypothesis, we established a cohort of 24 RM started on ART 9 days after intravenous infection with 5000 infectious units of SIVmac239M (Fig. 1a, **Supplementary Table 1**). PVLs peaked at 9- or 12-days post infection (dpi) with a geometric mean of 7 log_10_ RNA copies containing a median of 422 distinct barcodes per RM (Figs. 1b,c). After ART initiation, PVLs declined to <15 copies/ml in almost all RM by 15 weeks post-infection (wpi) and were below 1 copy/ml at most timepoints throughout the remainder of the 70-week ART treatment period (Fig. 1b). PBMC-associated and biopsy tissue vRNA and vDNA [including peripheral lymph node (pLN), mesenteric lymph node (mLN), spleen, liver, bone marrow (BM), duodenal (duo) and rectal mucosa], mirrored PVL with high vRNA and vDNA at day 12, with a subsequent on-ART decline of vRNA of 3–6 log_10_, compared to only 1–2 log_10_ for vDNA (Figs. 1d,e), consistent with previous observations of SIV on-ART dynamics^4,5^. At the end of the 70-week ART phase, the 24 RM were divided into 3 balanced groups based on peak PVL, time to viral suppression, number of viral blips, and overall virus levels in PBMCs and on-ART biopsies (**Extended Data Figs 1, 2**). Six RMs were necropsied on ART (to establish a baseline for virologic parameters during ART-suppressed infection) and 9 RM each were necropsied at day 5 and day 7 post-ART cessation to capture the predicted earliest rebound events. Among the RMs necropsied at day 5 and 7 post-ART, 2 of 9 and 5 of 9, respectively, manifested measurable rebound viremia (≥5 copies/ml) at necropsy (Fig. 1f). In the RMs with measurable plasma rebound, PVLs were less than 30 copies per ml at necropsy in 6 animals with the remaining RM showing more advanced plasma rebound at 4700 copies/ml. Barcode sequencing demonstrated 2–5 rebound barcodes in plasma of low PVL RMs, whereas 9 barcodes were identified in the plasma of the higher viral load RM (**Extended Data Fig. 3**). Of note, in a companion study using the same SIVmac239M/RM model, 16 RMs were necropsied at day 12 post-ART cessation to evaluate the dynamics of viral rebound in animals at later phases of rebound viremia (King, et al.).

In the 24 RMs studied here, an average of 93 tissue samples (range 86–123; **Supplementary Table 1**) across ~60 distinct tissue sites were collected and analyzed from each RM, including gastrointestinal (GI) tract, GI tract-draining LNs, non-GI tract-draining lymphoid tissues, and non-lymphoid tissues (using a consistent tissue collection protocol across all study RM; **Supplementary Table 2**), with the goal of sampling both major and minor sites known to harbor residual virus on ART^10,13,20^. Each collected sample was examined for overall SIV vRNA and vDNA with the vast majority of GI tract, GI tract-draining LNs and non-GI tract-draining lymphoid tissues showing detectable vDNA and most with detectable vRNA (**Extended Data Fig. 4**) at levels similar to those observed in on-ART biopsies (**Extended Data Fig. 2**). Both vDNA and vRNA were detected less frequently in non-lymphoid tissues, likely reflecting the lower proportion of potential viral target cells in these sites.

### Analysis of viral barcodes in tissues during ART defines the vRNA expression landscape during fully suppressive treatment

**Figure 2:**
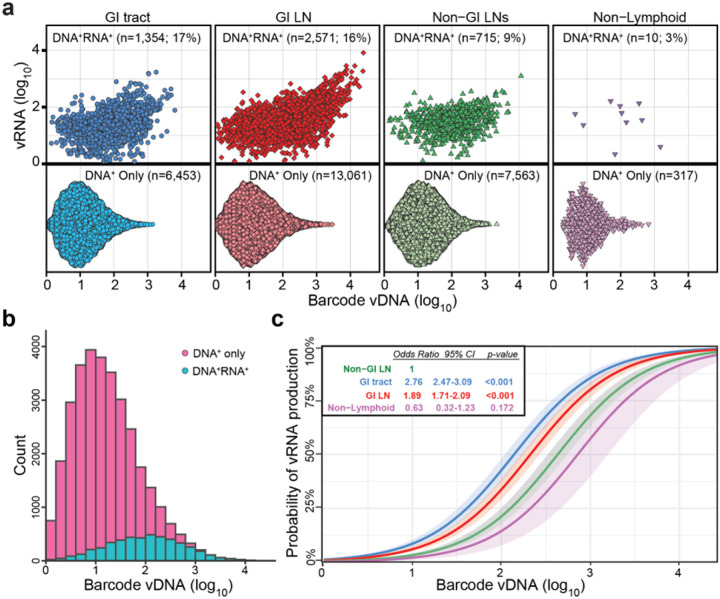
On-ART necropsy viral DNA and RNA comparisons per barcode overall and within each tissue group. **a**, vRNA (cDNA) and vDNA from individual samples with >50 available viral templates were sequenced to determine the viral barcode. Each barcode from each individual tissue sample is plotted per tissue group with barcodes expressing vRNA in only 3–17% of the total barcodes per tissue. **b**, Histogram (across all tissue groups) of the number of vDNA^+^ only counts compared to the vDNA^+^/vRNA^+^ counts based on the total vDNA count per viral barcode lineage showing that the more frequently a barcode is found in the vDNA, the more likely it is to also be found to be expressed as vRNA. **c**, The marginal effect of vDNA (log_10_) level stratified by type of tissue on the predicted probability of vRNA expression for a logistic regression model including animal-level random effects. For a given vDNA level, barcodes in GI tract tissue have the highest probability of being vRNA+, followed by GI tract-draining lymphoid tissues, non-GI LN, and non-lymphoid tissues. The shaded bands represent the 95% confidence intervals, indicating uncertainty around the estimated marginal effects, with odd-ratios for types of tissue relative to non-GI LNs shown in the figure inset, with p-values computed using the Wald-test.

Barcode sequencing was performed on vRNA- and vDNA-positive tissue specimens, with barcode-specific analyses focused initially on the 6 RM that were necropsied on ART. This sequencing showed the majority of detected barcodes across all types of tissue were vDNA+ only (i.e., without detection of matching barcode vRNA expression) (Fig. 2a,b). The percentage of barcodes expressing vRNA was higher in GI tract and in GI tract-draining LNs (16–17%), compared to non-GI tract lymphoid tissues (9%) and non-lymphoid tissues (3%). Part of this difference can be attributed to the observation that barcodes that are present at higher vDNA levels (more cells infected with a given barcode variant in the population), even with a fixed proportion expressing vRNA, are more likely to have sufficient numbers of these cells being transcriptionally active and therefore detected as vDNA+/vRNA+. Reflecting this, between 2 and 3 log_10_ levels of DNA detection, there is a notable shift in the population from vRNA expression likely originating from single cells (each with a distinct barcode) to apparent production of vRNA from multiple cells expressing the same barcode. This is most notable in the GI tract-draining LNs where proportionately more barcodes have vDNA levels exceeding 1000 copies compared to all other tissue groups (347 out of 15,632 vs 101 out of 16,412; 2-sided p<2E-16; two-proportion z-test; Fig. 2a). Importantly, mixed-effects logistic regression indicates that while barcode vDNA level is the strongest predictor of vRNA expression (p< 0.001; 2-sided Wald test), tissue type is also a significant explanatory variable, with barcodes in GI tract and GI tract-draining lymphoid tissues having significantly higher odds of being vRNA+ for a given vDNA level than barcodes in non-GI tract lymphoid tissues (p<0.001; 2-sided Wald test; Fig. 2c, **Supplementary Table 3**). Taken together, these observations suggest that at the level of barcode-defined clonotypic populations, the vDNA+ infected cell pool is either larger or more active or both in GI tract and GI tract-draining LNs than in other sites.

**Figure 3:**
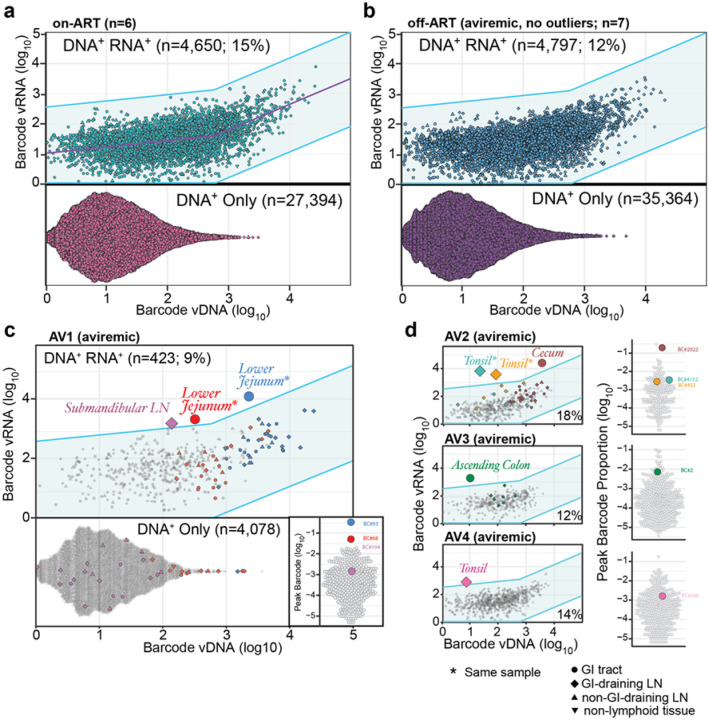
Detection of viral barcode lineages with vRNA expression enriched above on-ART RMs. **a**, The per barcode vDNA and vRNA levels across all tissues for each on-ART RM (n=6) reveal a piecewise linear regression (purple line) which was used to generate a 99% prediction interval (blue shaded envelope) for how much total vRNA is expected for a given vDNA level under conditions of full ART suppression. **b**, The vRNA and vDNA values show 7 of 11 off-ART, aviremic RMs were within the 99% PI. **c**, One off-ART, aviremic animal (AV1) had evidence of 3 distinct viral barcodes (BC.93; blue, BC.68; red, and BC.194; purple) outside the prediction interval in one of two distinct tissue pieces (lower jejunum and submandibular LN). Barcode 93 is the dominant peak plasma barcode which is also highly enriched in necropsy tissues. Barcode 68 was the second highest peak plasma barcode lineage and shows a highly enriched vDNA+ population in necropsy tissues with ~66% of tissues positive for vRNA. In contrast, Barcode 194 is not prominently represented in the peak plasma and shows outlier vRNA expression in a single necropsy tissue (submandibular LN). **d**, For the remaining 3 off-ART, aviremic RMs (AV2-AV4) the percentage of vDNA^+^, vRNA^+^ positive barcodes ranged from 12–18% with evidence of 1–3 outlier barcode lineages present at high peak plasma proportions and total necropsy tissue vDNA+ population (BC.2022; BC.4152; BC.452; AV3, BC.2) while BC.268 in AV4 is an outlier barcode despite a modest to low-level of overall viral burden at peak and at necropsy.

In order to develop criteria for detection of rare barcoded viral clonotypes contributing to initial viral outgrowth in tissues after ART cessation, the distribution of vRNA and vDNA expression levels per barcode measured across all tissues in the 6 RM necropsied on fully suppressive ART was plotted (Fig. 3a). A piecewise linear regression was used to define the relationship between the levels of vRNA expression and levels of vDNA per barcode. A 99% prediction interval (99% PI) was then determined for this distribution, defining the range of barcode vRNA expression expected for a given barcode vDNA level in RMs on fully suppressive ART (i.e., the maximum measured levels of vRNA expressed without active viral replication). As expected, the barcode-specific vRNA levels from the late-phase, pre-necropsy, on-ART tissue biopsies for all 24 RMs in the study fell within this 99% PI (**Extended Data Fig. 5**). As indicated above, the earliest post-ART stages leading to viremic rebound are pauci-clonal, and thus the transition from an ART-suppressed, non-replicative infection to active viral replication with only local tissue expansion would be expected to involve individual barcode clonotypes with a preferential increase in vRNA expression with, initially, little change in vDNA for those barcodes, given the potential for a single proviral vDNA to give rise to multiple logs of vRNA copies upon infection of new target cells, which typically would contain a single integrated viral genome each. Such events would be detected as outliers – individual barcodes with RNA expression exceeding the 99% prediction interval determined from the on-ART tissue samples.

### Identification of presumptive tissue origin sites of initial clonotypic post-ART rebound

We next analyzed tissue samples obtained using the same necropsy/tissue sampling protocol from the 11 aviremic RMs that were necropsied at 5 days (n=7) or 7 days (n=4) off-ART, each with PVL <1 copy/ml. As expected, given the limited time off ART and aviremic status, the per barcode vRNA and vDNA distribution from 7 of 11 aviremic RMs was indistinguishable from the distribution for RM necropsies on ART (Fig. 3b), suggesting that in the tissues examined in these 7 RMs, there had not yet been any measurable increase in expression of the vRNA of any individual barcode above the 99% PI determined for the on-ART group. However, in 4 aviremic, off-ART RM, 8 individual barcodes in 6 distinct tissue samples were identified that exceeded the 99% PI (Fig. 3c,d). The number of outlier barcodes detected per RM was 3, 3, 1 and 1 (AV1-AV4, respectively) all originating from the GI tract or GI tract-draining lymphoid tissues. Interestingly, 3 of these outlier barcodes (BC.93, BC.68, BC.2022) were identified near the top of the primary infection peak plasma virus barcode distribution, with overall high levels of vRNA expression in many necropsy tissues. On the other end of the spectrum, barcodes BC.194 and BC.268 were not dominant barcodes with no other vRNA+ tissue sites except for the noted outlier. Despite these differences in the distribution within barcode hierarchies for individual RMs, only 8 barcodes (of the over 4,500 barcodes detected in these 11 RMs) and 6 tissue sites (of the over 1,000 tissues analyzed) show evidence of vRNA levels higher than expected for viral expression only and thus likely reflect early off-ART viral replication with only local, within-tissue spread. However, since these RMs were aviremic at necropsy, the increased vRNA detected at these sites cannot be used to explicitly demonstrate a link between the measured tissue-based increases in barcode vRNA and systemic, viral rebound leading to measurable viremic rebound.

**Figure 4:**
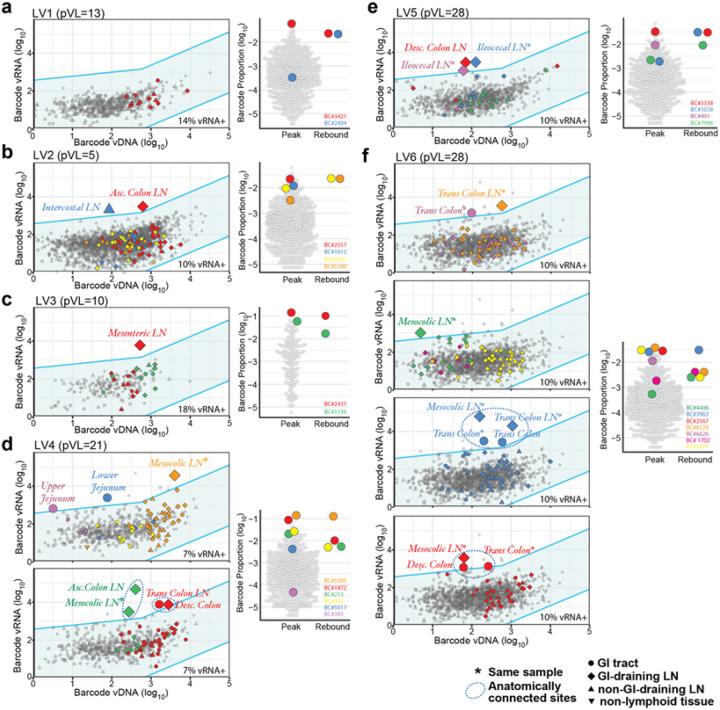
Off-ART, low viremic RMs demonstrate linkage between tissue outlier barcodes and rebound plasma. **a, b**, Two low-viremia RMs (LV1 and LV2) had evidence of two rebounding barcode lineages in plasma each, but none were identified as outliers in any analyzed necropsy tissue. **b**, LV2 showed evidence of two outlier barcodes in ascending colon LN (BC.2557) and an intercostal LN (BC.1012) but not in rebound viremia. **c**, LV3 had two distinct viral plasma rebound barcode lineages (BC.2431 and BC.1136), of which BC.2431 was also identified as a vRNA expression outlier in a mesenteric LN, linking this distinct tissue site with rebound plasma viremia. **d**, From LV4, 4 detectable barcode lineages were observed in rebound viremia (BC.5385, BC.1872, BC.213, and BC.834), 3 of which were also vRNA expression outliers in 1 or 2 tissues. **e**, LV5 had 3 detectable plasma rebound barcode lineages, of these, 2 (BC.3338 and BC.1078) were also found as vRNA expression outliers in a descending colon LN and in an ileocecal LN, respectively. Although barcode 401 (purple) was not identified in rebound plasma, it was enriched within the same ileocecal LN sample that gave rise to BC.1078. **f**, VL6 had 5 detectable rebounding viral lineages in plasma, 2 of these rebounding lineages (BC.8128 and BC.4406) were also found as vRNA expression outliers within a transverse colon LN and in a mesocolic LN, respectively. One additional rebounding lineage (BC.3963; blue) was the dominant lineage in rebound plasma and was found enriched in 4 distinct GI tract and GI tract-draining LNs. Two additional barcode lineages (BC.662; purple and BC.2567; red) were not found in rebound plasma but were identified as vRNA expression outliers in transverse colon and in three sites in the GI tract and GI tract-draining LNs, respectively. The frequency of all tissue barcodes that were vDNA^+^/vRNA^+^ ranged from 10–18%.

However, if outlier barcodes in specific tissue sites reflect local tissue events that are a precursor to post-ART viremic rebound, we predicted that the outlier barcodes in such tissues should overlap with the barcodes detected in peripheral blood in the RM with early post-ART viremia. Indeed, among the 6 RM with low-level post-ART viremia (LV1-LV6; <30 copies/ml rebound PVL), we detected 16 outlier barcodes in one or more than one tissue in 5 RM (LV2-LV6), of which 9 were also identified in the low-level early rebound viremia for 4 of the RM (LV3-LV6) (Fig. 4). Moreover, of the 18 barcodes detected in rebound plasma of LV1-LV6, half were also identified as outlier barcodes in one or more tissues examined. The remaining 9 rebound viremia barcodes had no identifiable tissue origin site, likely due to sampling limitations. For the 7 outlier barcodes that were detected in tissues but were not found in blood it is likely that these clonotypes had simply not yet reached a large enough replicating mass to spread into blood at measurable levels. Importantly, all but 1 of the 16 outlier barcodes detected in these low viremic RM were found in either GI tract or GI tract-draining lymphoid tissues. Furthermore, 12 of these 16 outlier barcodes were found in only one tissue site, while the other 4 outlier barcodes were found in more than one site that were anatomically linked within the GI tract or LNs draining these sites (dotted circles; Fig. 4d,f). Overall, in these 6 low-viremic RMs only 16 distinct tissues out of the nearly 600 examined (2.7%) had evidence of clonal viral replication and spread, highlighting the rarity of early rebound and demonstrating that early, limited off-ART viral replication with local within-tissue spread can produce low-level rebound viremia.

### Characterization of tissue origins and spread with subsequent clonal viral rebound.

**Figure 5:**
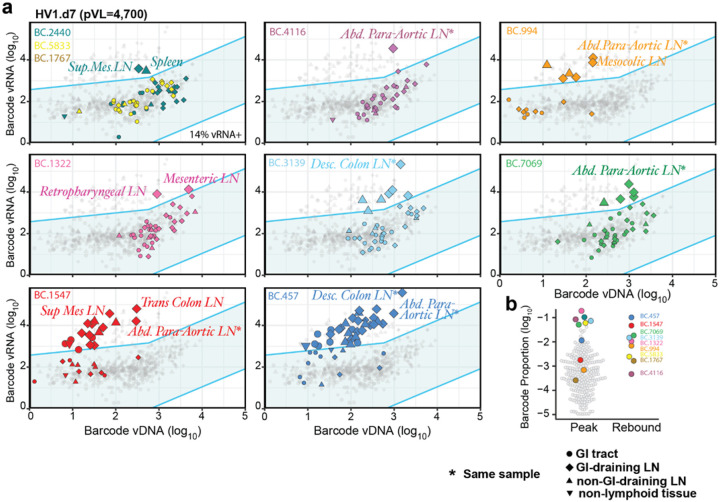
Off-ART high viremic RM shows evidence of off-ART local and distal replication and spread. **a**, RM HV1 had a rebound pVL of 4,700 copies/ml with 9 detectable rebounding barcode lineages in plasma and 8 barcodes showing outlier vRNA expression levels in 1 or more tissues (7 of these barcodes were also present in rebound plasma). 14% of all tissue barcodes were vDNA^+^/vRNA^+^. Barcode identified in rebound plasma but not demonstrated with outlier vRNA expression in any analyzed tissue (BC.5833; yellow and BC.1767; brown, only vDNA^+^) are shown in top left panel along with the only barcode lineage with outlier vRNA expression that not detected in rebound plasma (BC.2440, teal). GI tract samples are designated by circle, GI tract-draining lymphoid tissue are expressed at diamonds, non-GI-draining LN are displayed as up triangles, and non-lymphoid tissues as down triangles. **b**, The peak plasma barcode distribution compared to the rebounding, off-ART barcode hierarchy.

These observations of the earliest stages of viral rebound are consistent with the hypothesis that the outlier barcodes, enriched for vRNA expression, reflect local viral replication and tissue spread upon ART discontinuation, with subsequent spillover into the peripheral blood and local lymphatic spread, all of which would be expected to be a prelude to exponentially increasing viremia and distal (systemic) viral spread. Indeed, in one RM (HV1) that manifested more advanced rebound with a PVL of 4,700 copies/ml at necropsy (Fig.1, **Extended Data Fig. 3b**) there were overt signs of replication consistent with both local and distal viral dissemination (Fig. 5a). In this RM, a total of 9 barcodes were detected in rebound plasma, 7 of which were detected as outlier barcodes in 1 or more of the sampled tissues (BC.4116, BC.994, BC.1322, BC.3139, BC.7069, BC.1547, and BC.457), while only two rebounding lineages were not found as tissue outlier barcodes (BC.5833 and BC.1767) (Fig. 5a,b), likely because the sites of tissue origin of these barcodes were not analyzed. Interestingly, among rebounding barcodes, there was a direct relationship between the level of the rebounding barcode vRNA in blood and the number of tissues in which that barcode was found as an outlier. Note that the 2 barcodes with the highest representation in blood (BC.457 and BC.1547) showed outlier status in 32 and 17 diverse tissues, respectively, with a broad range of vRNA detected, with some tissues being far from the 99% PI boundary and others closer to this boundary. Intermediate viremic barcodes (BC.7069, BC.3139, BC.1322, BC.994) manifested outlier status in fewer, but still disparate tissues, whereas the lowest viremic barcode (BC.4116) was found as an outlier in only 1 tissue. Finally, one outlier barcode (BC.2440) was found in 2 tissues, but not in rebound plasma, consistent with a more recent viral reactivation and corresponding limited spread.

To identify the likely origin site in 3 RMs (LV4, LV6, HV1) with outlier barcodes found in more than one tissue (n=11 outlier barcodes), we determined if the tissue site with the greatest difference in barcode vRNA from that expected based on its vDNA level showed evidence of prolonged replication compared to other tissue sites. For these 11 barcodes, the adjusted vRNA level, e.g. the difference in measured vRNA compared to the on-ART predicted value (based on the corresponding vDNA level) was determined for each tissue in which it was an outlier. For each barcode, the tissues were rank-ordered based on their adjusted vRNA, and the differences in adjusted vRNA levels between consecutively ranked tissues were computed and used to determine a threshold difference level (99^th^ quantile) to distinguish sites of secondary spread from origin sites. A presumptive origin site (for which the adjusted vRNA distance to the next-highest site was above the threshold) could be identified for 6 of 11 barcodes, including 3 from HV1 (**Extended Data Fig. 6**). Thus, this single RM shows the entire spectrum of sequential reactivation and spread of individual barcode lineages ranging from clonotypes that have replicated and spread locally in tissue but not yet reached detectable levels in blood, clonotypes that replicated and spread in a single tissue site and have spilled into blood at low levels, and clonotypes that have extensively replicated and spread from initial sites to diverse distant tissues (consistent with secondary reseeding of tissues) with high levels in blood.

**Figure 6:**
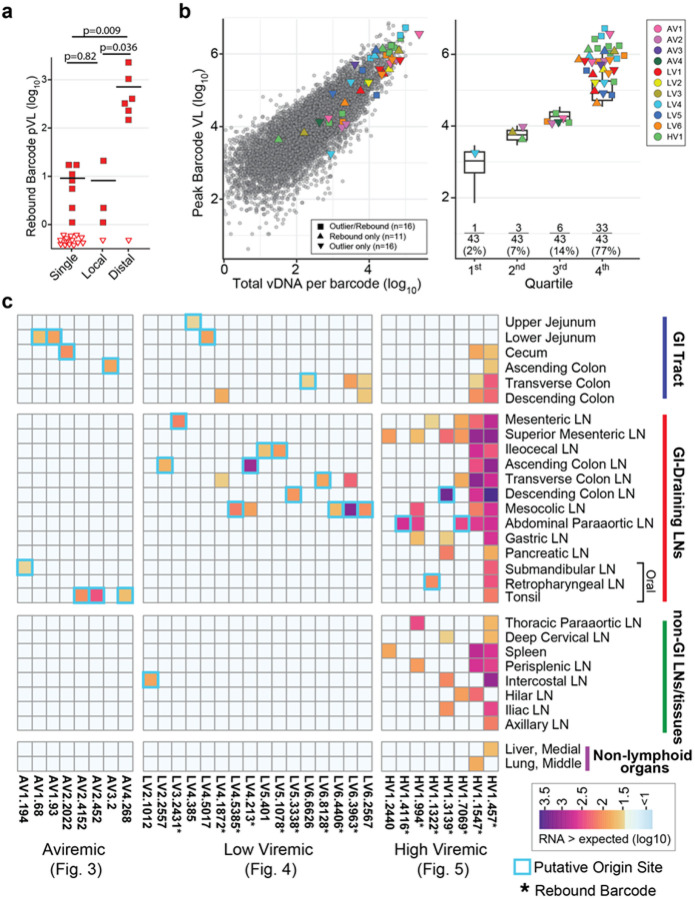
Outlier and rebound barcode lineages and origin sites are overrepresented in GI tract and GI tract-draining lymphoid tissues. **a**, The rebound PVL for each barcode found at a single site or with only local replication was significantly different from barcodes found spread to anatomically distal sites (Wilcoxon test, with Benjamini-Hochberg correction). **b**, The peak barcode levels are plotted against the total vDNA per barcode count with outlier and rebounding barcodes of interest highlighted and color coded by animal. The top peak plasma barcode proportions quartile contained 77% of all outlier and rebounding barcodes of interest. **c**, Heat-map shows all outlier barcodes above the 99% prediction interval, plotted by tissue sample type with the color values based on the level of vRNA above the expected value given its vDNA concentration. The tissues that represent rebound origin sites are outlined in cyan. There are 7 origin sites found within 5 GI tract tissues (upper and lower jejunum, cecum, ascending- and transverse-colon). For the GI tract-draining lymphoid tissues, 19 origin sites were detected from 10 different tissues (mesenteric LN, ileocecal LN, ascending colon LN, transverse colon LN, descending colon LN, mesocolic LN, abdominal paraaortic LN, tonsil, submandibular LN, and retropharyngeal LN). The non-GI LNs had evidence of a single origin site (intercostal LN). No origin sites were detected in non-lymphoid organs. Asterisk in column name indicates that the specific, outlier barcode was also found in rebound plasma.

These observations are consistent with sequential post-ART viral replication and spread, initially of just a few viral lineages with local spread within the origin site, followed by local (lymphatic) spread, and eventually to distal (hematogenous) spread with exponentially increasing rebound viremia. In this regard, it is interesting to note that when outlier barcodes are found in a single site, the majority (67%) are not detected in rebound viremia with the remaining 33% observed in plasma at levels of 1–18 RNA copies per ml (Fig. 6a). Locally spreading barcodes are more likely to become viremic, but still at very low levels (1–21 copies/ml). In contrast, barcodes with demonstrated distal spread show PVL levels above 150 copies/ml. These data suggest that by the time rebound viremia reaches levels that would be detected in the clinical setting (between 50–200 copies/ml), the process of viral rebound has already progressed beyond replication at the site of origin and has most likely also spread systemically.

### Initial viral rebound preferentially occurs in GI tract-draining lymphoid tissues.

Barcode RNA proportional representation at peak PVL during primary infection reflects levels of acute phase viral replication for individual barcode viral clonotypes and correlates with extent of total tissue seeding as measured by vDNA levels for all barcodes across all RMs (r=0.88, p<2×10^−16^; Pearson correlation). Each of the tissue outlier and/or plasma rebounding barcodes shown in Figs. 3–5 are highlighted within this overall distribution (Fig. 6b). Of note, 77% of outlier or rebounding barcodes detected derive from the top quartile of these barcode hierarchies (Fig. 6b). In fact, for RMs necropsied off-ART, mixed-effects logistic regression indicates that a 10-fold increase in the total tissue vDNA reservoir size for an individual barcode increases its odds of contributing to rebound nearly 12-fold (p<0.001; 2-sided Wald test) (**Supplementary Table 4**). However, barcode vDNA levels explain only about half of the variation (marginal R^2^ = 0.51), with 23% of outlier barcodes found much lower in the distribution (Fig. 6b), suggesting that tissue location-specific factors could influence the activation of much rarer clonotypes.

To visualize the tissue origins of rebound, a heat map of all 32 outlier barcodes in the aviremic, low viremic, and high viremic RMs was generated with the color scale indicating the individual barcode vRNA levels above the amount expected based on the amount of vDNA for that barcode with tissues meeting the criteria for rebound origin sites indicated by a cyan outline (27 of 32 outlier barcodes) (Fig. 6c, **Extended Data Fig. 6**). It is notable that with one exception (an intercostal LN in LV2), all other origin sites were in the GI tract (7 of 27; 26%) or GI tract-draining lymphoid tissues (19 of 27; 70%). Logistic regression indicates that across the tissues in which these 27 lineages were found, the odds of rebound originating in GI-draining LNs was 10-fold higher compared to non-GI draining LNs (p=0.022, 2-sided Wald test), whereas the barcode vDNA level in tissues was not a significant predictor of where rebound originated (**Supplementary Table 5**, **Extended Data Fig. 7**).

Importantly, the barcode itself is simply a marker to quantify distinct rebound events in tissues, allowing us to investigate predictors of rebound at the level of tissues rather than just within individual lineages. To this end, we extended our analysis to the 696 distinct tissue samples from which we obtained RNA and DNA barcode sequences of the 18 off-ART animals using a mixed-effects Poisson regression, which revealed a 10-fold higher incidence rate (IR) of barcode level rebound in GI-draining lymphoid tissues compared to non-GI LNs (p=0.025; 2-sided Wald test) while again, in contrast to the levels of individual barcodes, the total level of vDNA in individual tissues was not associated with rebound (**Supplementary Table 6; Extended data Fig. 8a**). Across the 1,717 sampled tissues from the 18 off-ART RMs, only 21 (1.2%) were identified as origin sites. Remarkably, 5 of these 21 rebound tissue origin sites had more than 1 rebounding barcode (Figs. 3–5), suggesting that local conditions in specific tissue sites likely also contribute to a milieu that favors initiation of viral rebound. Moreover, within the 21 rebound origin sites, the ranking of barcode DNA levels was predictive overall of which lineages rebounded (p<0.001, **Supplementary Table 7; Extended Data Fig. 8b**). However, in contrast to this overall pattern, several barcodes with low levels of DNA also contributed to rebound (**Extended Data Fig. 8b**), further suggesting a possible role of tissue micro-environments or cell-specific factors (e.g., integration site, activation state) contributing to off-ART rebound.

Overall, we have demonstrated that following ART release, a few individual barcode lineages begin actively replicating, most often in GI tract-draining lymphoid tissues, or the GI tract itself, leading to local and systemic spread of each lineage that culminates in rebound viremia. Taken together, the data indicate that both quantitative (the level of a given barcode in tissues) and qualitative (the type of tissue in which a given barcode is established) factors influence the probability of a given viral barcode clonotype to contribute to initial post-ART rebound.

## Discussion

The RCVR, virus which persists despite prolonged suppressive ART and can give rise to recrudescent, progressive infection when ART is discontinued, is the major barrier to an HIV cure. While various assays have been developed and used to quantify and characterize aspects of the RCVR on ART and the kinetics and clonal composition of viremic rebound after ART cessation have been described in detail in both humans and RMs^27^, the processes which connect the RCVR on ART to viremic rebound are not well understood. Yet, the fact that viremic rebound is temporally dispersed and oligoclonal despite the widespread distribution of cells harboring the RCVR indicates that tissues likely play a major role in the rebound process, implicating microenvironmental mechanisms in tissues that might be targetable for new approaches to achieve durable post-ART viral remission or cure. In order to study these potential mechanisms, the earliest phase of viral rebound, those first viral clonotypes emerging after ART discontinuation and spreading to and from new target cells, must be identified. We developed a statistically based criterion for detecting these initial sites of post-ART clonotypic viral spread and validated this criterion by demonstrating that barcodes of viruses showing evidence of local or regional replication post-ART (“vRNA outliers”) contribute to post-ART viremia. Indeed, among the 18 barcodes identified in the plasma of the 6 post-ART low viremic RMs, 9 were identified as rebounding barcodes in a single tissue or regionally linked tissues, a remarkable proportion that validates our vRNA “outlier” hypothesis and our experimental approach.

Not all barcoded viruses found in post-ART plasma were identified in sampled tissues, which is not surprising since even our extensive tissue sampling still represented only a relatively minor component of each animal’s total body lymphoid tissues. The implication is that the viremic barcodes not identified in sampled tissues are derived from sites that were not sampled. It is also not surprising that we identified locally spreading barcodes in individual tissues from some post-ART RM that were either aviremic or viremic with different barcoded viruses. In the former situation, these locally spreading barcoded viruses likely reflect early rebound founder sites that had not achieved sufficient replication to disseminate into blood by the time of necropsy, and in the latter situation, we would interpret these barcodes as reflecting later reactivating and spreading viral lineages (e.g., preceded by other barcoded viruses present in the rebound viremia) for which replication and spread was again interrupted by necropsy, prior to achieving viremia. On the other end of the spectrum, in an RM with more advanced viremic rebound (4,700 copies/ml), 7 of 9 rebounding barcodes in plasma were also found in tissues, with the barcodes showing the highest plasma levels showing disseminated replication and spread and those lineages at the lowest levels showing local replication and spread in one or two sites. These results are consistent with a temporal succession of viral clonotype local replication and spread: first in a single local tissue without viremia, continued spread in that tissue and possibly draining LNs with low viremia, and finally dissemination to multiple non-contiguous tissues with exponentially increasing viremia. This successive temporal process would be expected to continue for many viral lineages. Indeed, in a separate parallel study with necropsy of barcoded SIV infected RMs at day 12 post-ART, successive secondary rebounding lineages can be visualized, even as the initial lineages approach peak viremia (King, *et al*., submitted). Although this process has been shown using barcoded clonotype proportions in plasma rebound, to our knowledge, these studies provide the first data demonstrating this temporal progression in specific identified tissues, culminating in rebound viremia and recrudescent systemic infection of individual lineages reactivating at various times off-ART. Although this process has been shown using barcoded clonotype proportions in plasma rebound^4,24^, to our knowledge, these studies provides the first data demonstrating this temporal progression in specifically identified tissues, culminating in rebound viremia and recrudescent systemic infection of individual lineages reactivating at various times off-ART.

We identified 32 outlier tissue barcodes in study RMs, including 27 barcodes that met statistical criteria for presence in a potential or presumptive rebound origin site for that lineage (Fig. 6), allowing for statistically based conclusions about key characteristics of post-ART viral rebound biology. First, early rebound is typically detected in a single tissue in the absence of measurable viremia, but in some instances, replication in a single tissue or in a few anatomically contiguous tissues can be sufficient to achieve low but measurable levels of viremia (<30 copies/ml). In the single, higher viremic RM, distant spread (non-anatomically contiguous tissues) was associated with viremia at ≥150 copies/ml. If these associations hold true for HIV rebound in humans, the implication would be that by the time rebounding plasma virus is clinically detected between 50–200 copies/ml, the infection has already disseminated across the body. Second, almost 80% of the barcodes participating in initial tissue rebound derive from the top quartile of the total body barcode hierarchy. These highly represented barcodes are more likely to express vRNA on ART, possibly increasing the probability of their participation in initial events leading to viral rebound. Moreover, viruses with highly represented barcodes are more broadly distributed across the body and are therefore more likely to reside in tissues or tissue microenvironments that support viral rebound. However, it should also be emphasized that 23% of the time, viruses containing less represented barcodes, including lineages with little to no vRNA expression, can initiate tissue rebound, suggesting that tissue microenvironmental factors may contribute to reactivation and spread of less extensively distributed proviral barcodes. Indeed, the observation that 5 of the 21 identified rebound tissue origin sites had more than 1 rebounding barcode is consistent with specific tissue conditions favoring rebound initiation in these sites.

Finally, and most importantly, we found that independent of sampling, initial rebound was almost exclusively found in tissues associated with the GI tract, in particular GI tract-draining LNs such as intestine-draining mesenteric LNs. Indeed, our data suggest that initial rebound is 10-fold more likely in GI tract-draining LNs than non-GI tract-draining LNs. The immunologic importance of the gut microbiome has been repeatedly demonstrated^28,29^, including specifically in the setting of HIV infection where intestinal dysbiosis and microbial translocation have been documented, processes to which gut draining LNs are preferentially exposed, and which have been implicated as major drivers of systemic inflammation and immune activation, even on ART^30–32^. The innate immune stimulation resulting from microbial translocation could have multiple, non-mutually exclusive relevant effects on post-ART rebound, including creating immunologic conditions that 1) support the maintenance of a larger and/or more transcriptionally active virally infected cell pool, 2) decrease the innate and/or adaptive immune barriers to viral outgrowth and/or 3) as suggested above, provide specific triggers that promote virus expression leading to productive replication in the absence of ART^31,33–35^. Indeed, in the cohort of RMs studied here, barcode lineages in GI tract and GI tract-draining lymphoid tissues manifest significantly higher odds of being vRNA+ for a given vDNA level than barcodes in non-GI tract lymphoid tissues, suggesting these GI tract-draining LNs are constitutively more active than their non-GI tract counterparts. Interestingly, in a parallel study of rebounding RMs necropsied at day 12 (King, *et al*., submitted), the founder sites for secondary rebounding lineages (e.g., lineages well down in the temporal rebounding hierarchy) were still preferentially located in GI tract and GI tract-draining lymphoid tissues, even after the onset of systemic immune activation. These observations suggest that the mechanisms that underlie preferential initial rebound in GI-tract-associated tissues may persist through peak viral replication. While further work will be required to ascertain the nature of these mechanisms, this observation has important near-term implications for development of HIV cure therapies. First, given that these sites are difficult to access in humans, this observation should spur development of imaging or collection techniques focused on these LNs. Second, these data suggest that against usual assumptions, post-ART rebound is, like acute HIV infection, at least initially a process that predominantly occurs in GI tract and GI tract-associated lymphoid tissues, and that interventions designed to intercept and interrupt post-ART rebound must be designed, formulated and delivered in a way that ensures activity in these sites. This is not to say that non-GI tract-associated sites are irrelevant for rebound, but clearly, interventions lacking activity in these sites would not be expected to be effective. Moreover, even an initial suppression of tissue rebound in GI tract-associated sites, delaying viremia for days to weeks, might facilitate immune control of infection, potentially leading to long-term remission^36,37^.

We acknowledge that this study uses a reductionist animal model and study design that does not capture all of the subtleties and complexities of human infection with HIV. While SIV infection of RM authentically recapitulates many key aspects of the pathogenesis of HIV in humans^38^, host species differences do exist. Moreover, this study employed SIVmac239M, a virus stock that uses a unique barcode tag in an otherwise isogenic and phenotypically equivalent virus to enable tracking of chains of viral infection events originating from individual virus variants, while obviating viral phenotypic differences as a potential confounding variable. While this critical feature does not capture the genotypic and phenotypic diversity of the complex swarms typically present in individuals with HIV infection, particularly if they started ART later in the course of their infection, this model does allow for the ultra-sensitive and unambiguous identification of each individual lineage within an animal and for each tissue sampled. Similarly, initiation of ART in this study at a precise uniform time in acute infection allowed the establishment of well-calibrated RCVR levels, based on characterization in previous studies in the model^4,24^, and although this does not reflect the typical timing of ART initiation in most humans, immediate ART treatment is the recommended treatment guideline^39,40^. A consequence of early ART initiation and just over a year of treatment before ART discontinuation is that the RCVR may still be undergoing decay^4,24,41^ and remains genetically intact^42^. This is in contrast to the accumulation of defective proviral genomes in individuals after extended periods of ART – often decades^43,44^. Additionally, early ART initiation and SIVmac239 resistance to antibody neutralization^45^ obviate the development of autologous neutralizing antibody as a potential confounding factor influencing viral rebound dynamics, while such antibodies have been shown to be an important consideration in people living with HIV^46^. Finally, while we have shown that expanded clones of infected CD4^+^ T cells can be established with early initiation of ART following SIV infection^47^, we did not characterize this aspect of the RCVR for this study and it is likely that clonal expansion is more limited in this model than seen in people with HIV infection on long-duration ART^48–50^.

The virology and immunobiology underlying local tissue-level processes and events leading to post-ART rebound in SIV-infected RM is likely similar for people living with HIV, in particular our fundamental finding that a few individual lineages begin actively replicating, most often in GI tract-draining lymphoid tissues with tissue-specific factors influencing the probability for a given viral clonotype to contribute to post-ART rebound. This general biology might be confirmed in people with imaging or other non-invasive means in subjects undergoing analytical treatment interruption. Moreover, the RM model itself, the barcode data, and the approach reported here will allow for a tissue-based, detailed analysis of initial rebound sites, providing insight into the mechanisms promoting or restricting post-ART viral rebound, and thereby informing the development of therapeutics that can exploit these mechanisms to achieve off-ART viral remission.

## Methods

### Animals.

A total of 24 purpose-bred male rhesus macaques (*Macaca mulatta*) of Indian genetic background, aged 2.5–4.7 years at study start, were used for these experiments (Supplementary Table 1). They were determined to be specific pathogen free, as defined by being free of cercopithecine herpesvirus 1, D-type simian retrovirus, simian T-lymphotropic virus type 1, and *Mycobacterium tuberculosis*. MHC-1 genotyping for protective MHC alleles (*Mamu*-A*01, *Mamu*-B*08 and *Mamu*-B*17; **Supplementary Table 1**) was performed by sequence-specific PCR as described previously^51^. Animals were born and housed at the Oregon National Primate Research Center following standards established by the Center’s Institutional Animal Care and Use Committee and the National Institutes of Health Guide for the Care and Use of Laboratory Animals. Prior to study initiation/infection, all RM received a multimodal therapeutic regimen to eliminate common gastrointestinal pathogens as previously described^52^. RM were inoculated intravenously with 5000 infectious units of barcoded SIVmac239M^4,24^ before starting daily ART as previously described^53^ [subcutaneous injections of 5.1 mg/kg/d tenofovir disoproxil, 40 mg/kg/d emtricitabine, and 2.5 mg/kg/d dolutegravir in a solution containing 15% (v/v) kleptose at pH 4.2] 9 days post-infection through at least 70 weeks post-infection. The viral dose and timing of ART start was calibrated based on past data in the model to provide a saturated reservoir as well as high resolving power for identifying individual barcode reactivation events, while not impacting acute infection viral dynamics^4,24^.

### Tissue sampling.

Tissue biopsies included bone marrow (collected from iliac crest or humerus), peripheral lymph nodes (collected from axillary or inguinal sites), mesenteric lymph nodes (collected from colonic mesentery), spleen and liver (collected as laparoscopic pinch biopsies), and duodenum and rectum (collected as endoscopic pinch biopsies)^54,55^. At necropsy, all efforts were made to collect lymphoid tissues and intestines comprehensively while representative samples were collected from other tissues of interest (Supplementary Table 2). All detectable lymph nodes (>~2mm in diameter) were collected from at least 20 pre-defined sites, with each lymph node collected individually. Spleen was divided into 3 mm sections orthogonal to the major axis. Bone marrow was collected from each femur. Thymus was divided into 4 sections. Small intestine was divided into duodenum (rostral-most 8 cm), jejunum and ileum (caudal-most 8cm) while large intestine was divided into cecum, ascending colon, transverse colon descending colon and rectum based on macroscopic morphological features. Each section of the intestines was further divided into 4 cm-long segments. Representative samples were collected from other tissues, including brain, spinal cord, heart, kidney, liver, lung, urinary bladder, prostate, testes, seminal vesicles, visceral fat, nasal mucosa and oral mucosa. Tissue samples for virological analysis were collected in either Lysing Matrix D (MP Biomedicals Inc; Irvine, CA; PBMC, tonsil, lymph nodes, bone marrow, spleen, thymus, adenoids, bronchoalveolar lavage and spinal cord) or Precellys lysing kit “Tissue Homogenizing CK28–15 ml” (Bertin Technologies, Montigny-le-Bretonneux, France; intestines, oral and nasal mucosa, lung, male reproductive tract, brain, liver, kidney, heart, fat) and flash-frozen immediately after collection.

### Viral detection assays.

Levels of plasma SIV RNA and vDNA and vRNA from cell pellets/tissues were determined essentially as described previously, using assays targeting a gag amplicon^56,57^.

### Barcode sequencing.

RNA and DNA for sequencing was obtained from samples extracted for vRNA and vDNA quantitation assays. Complementary DNA (cDNA) was generated with Superscript III reverse transcriptase (Invitrogen) and an SIV-specific reverse primer (Vpr.cDNA3). Prior to sequencing, PCR was performed with VpxF1 and VprR1 primers containing either the F5 or F7 Illumina adaptors with a unique 8-nucleotide index sequence for multiplexing. PCR was performed using High Fidelity Platinum Taq (ThermoFisher). The multiplexed samples were sequenced on a MiSeq instrument (Illumina) and analyzed as previously described^4,24^. Because elevated vRNA expression per vDNA level is used as a signal of active viral replication in subsequent analyses, to avoid false positives resulting from under-quantification of vDNA due to possible inhibition in the PCR reaction, we multiplied the quantified number of SIV DNA copies in each sample by the ratio of the estimated to the quantified sequencing input. Because RNA is less likely to be inhibited, and to avoid false positive signals of active RNA replication, we did not adjust RNA quantities even when the estimated sequencing input was higher than the quantified levels. To obtain the absolute SIV DNA and RNA copies per barcode in each sampled tissue, we multiplied the barcode proportions by the total (barcode-adjusted) SIV DNA or (original) RNA quantity in each sample, respectively. For subsequent analyses at the barcode level, only samples for which we obtained both RNA and DNA barcode sequences were retained to ensure that all observations were directly comparable across samples and animals. Finally, only barcode sequences that matched exactly those found in each RM’s peak (day 12 post infection) distribution were used.

### Statistical Analysis.

The Friedman rank sum test was used to compare PBMC vDNA and vRNA levels across all sampled time points, with the paired Wilcoxon-signed rank test used for pairwise comparisons between consecutive time points (12 dpi vs 21 wpi and 21 wpi vs 66 wpi), with values below the limit of detection (LOD) set to the LOD value (0.004 cp/10^6 cell equivalents). The two-sided Kruskal-Wallis test was used to compare animals between the three different experimental groups (on-ART, off-ART 5d, and off-ART 7d) in terms of peak plasma viral load, frequency of viral blips, and PBMC vDNA and vRNA at each sampled time point. Additionally, the log-rank test was used to compare the Kaplan Meier survival curves of time to suppression between the experimental groups. Finally, the two-sided Kruskal-Wallis test was used compare the cell-associated vDNA and vRNA obtained from different tissue biopsies between the three experimental groups at each biopsy time point. All statistical analyses were performed in R version 4.3.1 and Prism 10.4.1.

### Identifying active viral replication based on higher-than-expected on-ART RNA expression.

To determine how on-ART baseline RNA expression (per barcode per sample) varied as a function of DNA quantity, we fit a piecewise linear regression using the R package “segmented” with a single unknown breakpoint to the set of RNA+DNA+ barcodes (greater than 1 SIV RNA and 1 SIV DNA copy) across all samples from all 6 animals necropsied on-ART. The data was log_10_-transformed, and the regressions were weighted based on the DNA quantity due to increased precision of larger values. Davies’ test was performed to confirm a non-zero difference in slope between the two segments. We estimated the expected range of RNA expression for a given DNA level on-ART by constructing a 99% prediction interval (PI) for the fitted model. For all animals necropsied after ART discontinuation, we then identified all outlier data points with RNA expression above the on-ART 99% PI as putative sites of active viral replication..

### Identifying rebound origin sites.

Barcodes identified in rebound plasma that had a single outlier site indicative of active viral replication were deemed to have also rebounded from that site. To identify the putative rebound origin site for barcodes that were RNA-outliers in more than one tissue sample, we looked for a signal of delayed viral replication consistent with spread from the site of predominant RNA expression to other outlier sites. Specifically, we assumed that after rebound occurs at a single site, the virus will replicate at that site for some time before spreading to related tissues or disseminating broadly via the lymphatics, resulting in bigger differences in the level of replication between the origin site and the secondary site of replication, than between distinct secondary sites, which may have started replicating at around the same time. For each outlier, we first determined how much vRNA was expressed above the expected level based on the on-ART linear piecewise model fit, i.e., adjusted vRNA = observed vRNA (log_10_) – predicted vRNA (given the level of vDNA). For each barcode, the outlier sites were rank ordered based on their adjusted vRNA levels, and the difference in adjusted vRNA was computed between consecutive sites. We used the 99^th^ quantile of adjusted vRNA differences between secondary sites (rank > 1) as the threshold level for discriminating delayed replication consistent with replication from the origin site.

### Regression analyses.

Regression analyses were performed in R using the lme4 package for mixed effects and the geepack package for generalized estimating equations. All models investigated the effect of vDNA level on the outcome (probability of RNA expression or probability or incidence of rebound), with a subset of models additionally including tissue type. For models with more than one predictor of interest, we first investigated the effect of each covariate on the dependent variable independently and compared the models against each other based on the AIC score and against the null model (containing just the intercept) by the likelihood ratio test. If both covariates were significantly associated with the outcome (based on the asymptotic Wald test), we investigated if the full model containing both covariates explained the data better than the best-fitting single covariate model using the likelihood ratio test.

#### Model 1: Probability of RNA expression per barcode per sample

We performed mixed effects logistic regression analyses to investigate the effect of 1) the DNA level (log_10_) per barcode per tissue sample, 2) the tissue type of each sample, and 3) both covariates together, on the probability of RNA expression in the 6 on-ART animals. Clustering of observations within individual animals was accounted for by including a random effect on the intercept (assumed normally distributed with a zero mean).

#### Model 2: Probability of rebound per barcode

We performed mixed effects logistic regression analyses to investigate for the 18 off-ART animals whether a barcode’s total DNA across all sampled tissues was predictive of whether it rebounded (i.e. the barcode was an outlier based on the on-ART 99% PI in any tissue sample and/or was detected in rebound plasma viremia). Clustering of observations within individual animals was accounted for by including a random effect on the intercept (assumed normally distributed with a zero mean).

#### Model 3: Probability of rebound per sample within barcodes with identified origin sites

For the 27 barcoded lineages across the 18 off-ART animals for which we could identify presumptive rebound origin sites, we investigated whether tissue type or the barcode’s vDNA (log_10_) level in individual tissue samples were predictive of whether they were rebound origin sites. We again performed logistic regression analysis using GEE, accounting for clustering of observations within distinct barcoded lineages using an independent correlation structure.

#### Model 4: Incidence of rebound per tissue sample

We performed mixed effects Poisson regression analysis to investigate for the 18 off-ART animals whether either the total vDNA level of a tissue or tissue type were predictive of the rebound incidence rate per tissue. Clustering of observations within individual animals was accounted for by including a random effect on the intercept (assumed normally distributed with a zero mean).

#### Model 5: Probability of rebound per barcode within origin sites

For the 21 presumptive tissue origin sites across the off-ART animals, we investigated whether the vDNA level of individual barcodes were predictive of their probability of rebounding using logistic regression in the generalized estimating equations (GEE) framework, accounting for clustering of observations within distinct tissue origin sites. We used independent correlation structures in the models because more complex working correlation matrices did not improve quasi-likelihood under independence model criterion (QIC) scores.

## Supplementary Material

Supplementary Files

This is a list of supplementary files associated with this preprint. Click to download.

- supplementary.docx

## Data Availability

All data supporting the findings of this study are available within the paper, its Supplementary Information, including the Data Sheet.
